# Measurement of respiratory–swallowing coordination using an oronasal facemask in healthy individuals

**DOI:** 10.1113/EP092025

**Published:** 2024-09-12

**Authors:** Elizabeth Cross, Esther Guiu Hernandez, Phoebe Macrae

**Affiliations:** ^1^ Rose Centre for Stroke Recovery and Research University of Canterbury Christchurch New Zealand

**Keywords:** deglutition, dysphagia, oronasal respiration, respiratory–swallowing coordination, swallowing

## Abstract

Respiratory–swallowing coordination (RSC) is well established as an essential airway‐protective mechanism. Previous studies have used nasal airflow and/or kinematic rib cage and abdominal measures to assess respiration surrounding swallowing, meaning that the direct influence of oral respiration on RSC remains unknown. This study used a partitioned oronasal facemask to compare respiratory phase patterns measured using isolated nasal airflow with those measured using combined oronasal airflow during non‐ingestive and ingestive swallowing tasks. Twenty‐four healthy individuals with no respiratory or swallowing disorders were assessed at rest and during cued dry, 10 mL water, continuous drinking and cracker swallowing tasks. Respiratory phase patterns were determined for discrete swallows using the nasal and combined oronasal channels separately. There was variable agreement between respiratory phase patterns according to the nasal and oronasal channels across swallowing conditions. The frequency of exhale–swallow–exhale, inhale–swallow–exhale and exhale–swallow–inhale patterns increased by 2%–3% each with the addition of oral flow data to nasal data, whereas the prevalence of inhale–swallow–inhale and ambiguous patterns decreased. This suggests that estimates of respiratory phase patterns are altered minimally by inclusion of oral respiratory estimates in a healthy sample. There were several additional findings of note, including lower within‐participant, within‐session trial consistency (test–retest reliability) than expected, suggesting high variability in respiratory phase patterns across trials. Additionally, data showed evidence of swallowing non‐respiratory flow at the beginning and end of the respiratory–swallowing pause, moving in both inward and outward directions, potentially expanding current understanding of swallowing non‐respiratory flow. Further in‐depth physiological investigations are required to improve understanding of these findings.

## Abstract (highlights)

- **What is the central question of this study?**
  What is the impact of mouth breathing on the frequency of respiratory phase patterns for respiratory–swallowing coordination?
- **What is the main finding and its importance?**
  Added oral flow appeared to alter respiratory phase patterns minimally in healthy individuals. This was probably attributed to increased clarity of the respiratory flow signal, aiding interpretation of respiratory data. This suggests that mouth breathing might impact measurement of respiratory phase patterns. These findings might have implications for the methods used to assess respiratory phase patterns and broader respiratory–swallowing coordination, particularly in patient populations prone to mouth breathing.

## 1 INTRODUCTION

Respiratory–swallowing coordination (RSC) plays a crucial role in protecting the airway during ingestion of food and fluids. One aspect of RSC, respiratory phase patterning, is characterized by the position of swallowing within the respiratory cycle, i.e., mid‐expiration (exhale–swallow–exhale; EX‐EX), mid‐inspiration (inhale–swallow–inhale; IN‐IN) or at the junction between expiration and inspiration (exhale–swallow–inhale [EX‐IN] or inhale–swallow–exhale [IN‐EX]). Data suggest predominance of EX‐EX (71%–79%) during discrete bolus swallows (Martin‐Harris et al., 2005; Wheeler Hegland et al., 2009), followed by IN‐EX (Martin‐Harris et al., 2005), although this can vary depending on factors such as bolus volume (Hopkins‐Rossabi et al., 2019; Lederle et al., 2012) and during sequential swallows (Dozier et al., 2006; Wheeler Hegland et al., 2011). Expiration after the swallow is considered optimal (Martin‐Harris et al., 2023) and is thought to prevent bolus residue from entering the trachea and to clear residue from the entrance of the airway, thus acting as an airway‐protective mechanism.

Investigations of RSC have typically measured nasal airflow (Brodsky et al., 2012; Gross et al., 2009; Martin‐Harris et al., 2005, 2003; McFarland et al., 2016) and/or rib cage and abdominal movement (Gross et al., 2009; McFarland et al., 2016; Wheeler Hegland et al., 2011, 2009) to estimate respiration during swallowing tasks, with most studies using nasal airflow measures (Hopkins‐Rossabi et al., 2019). Although many healthy individuals breathe primarily through the nose (Leiberman et al., 1991; Nascimento et al., 2019; Niinimaa et al., 1981), simultaneous oronasal breathing is known to occur in others (Nascimento et al., 2019; Niinimaa et al., 1981), with higher rates of mouth breathing occurring in conditions such as chronic cough (Vertigan et al., 2020), vocal fold dysfunction (Vertigan et al., 2020) and obstructive sleep apnoea (Koutsourelakis et al., 2006; Nascimento et al., 2019). Reliance solely on nasal flow for estimating respiration might misrepresent RSC data in individuals who are prone to mouth breathing. This is supported by reports of lost data during periods of mouth breathing in a study assessing RSC in chronic obstructive pulmonary disease (Gross et al., 2009).

An oronasal facemask has been used in sleep studies to assess respiratory airflow from the mouth and nose simultaneously (Fitzpatrick, Driver et al., 2003; Fitzpatrick, McLean et al., 2003; Kukwa et al., 2014; McLean et al., 2005). A small number of RSC studies have used an oronasal facemask (Boden et al., 2009; Hardemark Cedborg et al., 2009; Paydarfar et al., 1995) but have not investigated the distinct influence of oral respiration on respiratory phase patterns. No known studies have examined the influence of oral respiratory flow on respiratory phase estimates. The aim of this study was to determine the influence of oral respiratory flow on respiratory phase estimates in healthy participants using a fully enclosed oronasal facemask with partitioned sections for the mouth and the nose during a range of non‐ingestive and ingestive swallowing tasks.

## 2 MATERIALS AND METHODS

### 2.1 Ethical approval

Ethical approval was granted by the University of Canterbury Human Research Ethics Committee (HREC 2022/34/LR‐PS). The study conformed to the standards set by the *Declaration of Helsinki*, with the exception of registration in a database.

### 2.2 Participants

Healthy participants who were >18 years of age were recruited. No upper age limited was enforced. All participants provided informed consent prior to their involvement in the study. Individuals with a reported history of swallowing impairments or respiratory conditions were excluded from the study. Three sessions were completed within 1 week, always with 1 day in between (i.e., sessions usually occurred on Monday, Wednesday and Friday of the same week). This was done to allow for assessment of test–retest reliability (consistency) of measures across sessions. The participants were not blinded to the aims of the study. The only exception to this was that, during the rest breathing period, participants were not expressly told that their swallowing would be assessed; this was to allow assessment of spontaneous swallows. Participants were simply informed that their breathing would be assessed during the rest period.

### 2.3 Instrumentation

A partitioned oronasal facemask (dual port silicone rubber face mask, Hans Rudolph, inc.) was used. The facemask was adapted to allow placement of a straw into the mouth. The straw was occluded at the external end when not in use to prevent extraneous airflow. Each port (nasal and oral) of the mask was connected to a filter (SureGard®, Bird Healthcare) and 300 L/min spirometry flow head (MLT300L, AD Instruments). The flow heads were each connected to a spirometer pod (FE141, AD Instruments) and calibrated using a 3 L calibration syringe (Hans Rudolph). Spirometry signals were recorded in PowerLab® (PowerLab 8/35‐2264, AD Instruments; sampling rate 100/s, range 500 mV) and analysed in LabChart® (v.8, AD Instruments) using the spirometry extension (Spirometry Analysis Software, v.2.5.3, AD Instruments). A pre‐set low‐pass analog filter of 100 Hz was used. Submental surface electromyography (sEMG; Trigno™ Wireless EMG System, Delsys) was used to supplement respiratory signals suggesting the occurrence of swallowing. The sEMG was bandpass filtered between 10 and 500 Hz (De Luca et al., 2010; Stepp, 2012) and could be viewed simultaneously with spirometry signals to ensure that swallowing and the associated pause were related.

### 2.4 Procedure

After preparation of the skin with an alcohol wipe, the sEMG electrodes were placed underneath the chin at the midline overlying the submental muscles. An earth electrode was placed on the left collarbone. The facemask was then fitted and secured to the head using a headpiece that was clipped to each corner of the mask. This was adjusted to ensure a consistent seal between the facemask and the skin. Additionally, the flow heads were suspended from a rod above the participant to relieve weight and to encourage consistency of posture.

Five different swallowing conditions were completed, namely spontaneous, dry, 10 mL water, continuous drinking and cracker. The participant sat at rest for 5 min, during which spontaneous swallows were assessed. The rest period was always completed first to minimize the participant's awareness of the swallowing tasks and, therefore, to increase the likelihood of truly spontaneous swallows occurring in this period. After completion of the rest period, the participant performed five dry swallows. Dry swallows were always completed after the rest period, hence they were not impacted by the following ingestive tasks. Dry swallows were cued using the instruction, ‘swallow when you're ready, and raise your hand when you swallow’. The instruction of ‘swallow when you're ready’ was used with the aim of eliciting the most natural swallowing pattern for the participant. The ingestive swallowing tasks were completed in a counterbalanced order across participants, after the non‐ingestive swallowing tasks. For water swallowing tasks, the straw was unplugged and inserted into the mouth. This was placed into a position that was comfortable for drinking, as indicated by the participant. For the two 10 mL water swallowing trials, a syringe was clipped to the external end of the straw, through which 10 mL of water was delivered into the mouth. The participant was instructed to ‘swallow the water when you are ready, and raise your hand when you swallow’. For the continuous drinking condition, a 150 mL cup of water was supplied to the participant. The participant held the cup during the trial and drank the water through the straw. They were instructed to ‘drink this as is comfortable, until you have finished the waterʼ. For the two cracker trials, the researcher unclipped the facemask and presented half an Arnotts Salada™ cracker segment to the participant. The participant was instructed to hold the cracker in the mouth without chewing until the mask had been re‐secured. Once this was complete, they were instructed to ‘chew the cracker, swallow when you're ready, and raise your hand when you swallowʼ. The researcher documented hand raises (dry, 10 mL and cracker), and observed laryngeal movement and audible swallowing‐related noise (continuous drinking) as a comment in LabChart®, which were used to supplement respiratory and sEMG signals.

### 2.5 Data analysis

Data analysis was completed by two raters; the primary rater was the same person who led all data collection sessions, and the second rater was a trained assistant. Neither of the assessors/raters were blinded to the aims of the study. Respiratory phase patterns were analysed separately from the nasal and combined oronasal respiratory flow channels. Oronasal flow was estimated by summing the nasal and oral flow channels in LabChart®. A positive polarity waveform (i.e., above zero) represented inspiration, and a negative polarity waveform (i.e., below zero) represented expiration. There was no prespecified threshold (amplitude or temporal) at which the airflow signal was considered to represent inspiration or expiration; instead, airflow was counted as respiratory if its amplitude was similar to the typical level of airflow for the individual. Any waveform that was subjectively close to zero or that did not align with the expected amplitude (based on surrounding airflow) was subjectively considered to be swallowing non‐respiratory flow (SNRF) or artefact. Occurrence of swallowing was determined based on the following criteria: a burst in sEMG activity coinciding with a plateau (zeroing) of the respiratory waveform, indicating cessation of breathing (for spontaneous swallows); this was supported by a participant's hand raise for cued dry swallows, 10 mL swallows and cracker swallows, or observation of swallowing by the researcher for continuous drinking. The first two spontaneous swallows to occur during the rest period were extracted for the analysis, as were all five dry swallows, the first three swallows to occur during continuous drinking, and two each of 10 mL and cracker trials. During analysis, both raters were aware of the swallowing conditions and channel (nasal or oronasal) from which each trial came.

**FIGURE 1:**
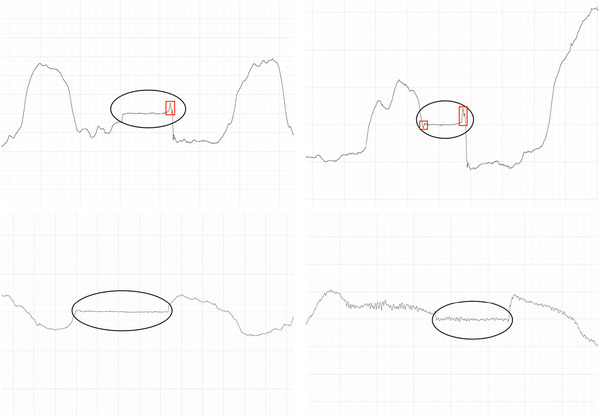
Respiratory phase patterns including exhale–swallow–exhale (EX‐EX; top left), inhale–swallow–exhale (IN‐EX; top right), exhale–swallow–inhale (EX‐IN; bottom left) and inhale–swallow–inhale (IN‐IN; bottom left). A negative‐polarity waveform indicates exhalation, whereas a positive‐polarity waveform indicates inhalation. The respiratory‐swallowing pause is circled in black. In the top left and top right images, swallowing non‐respiratory flow (SNRF) can also be observed (within red squares).

Swallows were categorized into the following five patterns (Figure 1): EX‐EX, IN‐EX, EX‐IN, IN‐IN and cannot judge (CNJ). Respiratory phase patterns were determined based on flow immediately before and immediately after the respiratory‐swallowing pause. As a result, the time frame in which they were observed could be slightly different according to the nasal and oronasal channels (due to differences in respiratory‐swallowing pause durations between conditions), although no clear differences were observed. If no clear respiratory phase was observed within 3 s either side of the respiratory‐swallowing pause, the trial was coded as CNJ. Raters were able to adjust the scaling on LabChart as required to analyse the respiratory waveform clearly. Additionally, they were able to review the entire recording to observe the typical respiratory patterns of the participant, to inform their decisions. Analysis of respiratory phase patterns was completed separately for the nasal and oronasal channels. Only the channel currently being analysed was visible (e.g., the oronasal channel was not visible when analysing the nasal channel and vice versa) to prevent the rater's decision being influenced by the other channel.

**FIGURE 2:**
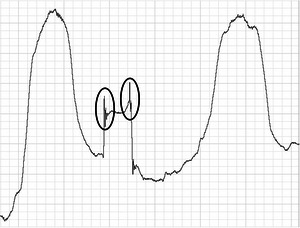
Example of swallowing non‐respiratory flow (SNRF) seen at the beginning and end of the respiratory‐swallowing pause. The SNRF events are circled in black.

Occurrence of apparent swallowing non‐respiratory flow (Figures 1 and 2), i.e., air flow detected that is not considered respiratory in nature (Brodsky et al., 2012), was recorded using a binary presence/absence criterion. Similar to respiratory flow patterns, there was no prespecified threshold (pertaining to amplitude or duration) with which to distinguish between SNRF and respiratory flow. As a result, presence of a SNRF event was determined based on subjective interpretation of the waveform. When deemed present, further information was recorded regarding its position within the respiratory‐swallowing pause (onset‐pause or offset‐pause) and direction of airflow (i.e., inward or outward).

Despite plans to compare across sessions, only data from the second data collection session were analysed initially, in order to assess intra‐ and inter‐rater reliability. Instead of analysing the first session, the second session was selected to minimize the influence of novelty of the facemask on respiratory behaviour. Intra‐ and inter‐rater reliability were found to be poor. Additionally, observations were made of inconsistent respiratory phase patterns within individuals, likely influenced by the poor reliability. As a result, conducting further between‐session comparisons was thought to be of little meaning. As such, data from the two remaining sessions were not analysed.

The full session 2 dataset was analysed by E.C. A total of 28 trials from each swallowing condition were re‐analysed by E.C. and another trained research assistant for intra‐ and inter‐rater reliability. Both raters had similar amounts of experience with respiratory phase analysis.

### 2.6 Statistical analysis

Intra‐ and inter‐rater reliability, agreement between nasal and oronasal channels, and within‐participant test–retest reliability were all calculated using Cohen's unweighted κ (*P *= 0.05, 95% confidence interval [CI]) and percentage of agreement. This was completed for all swallows combined, and separately for each swallowing condition (spontaneous, dry, 10 mL, cracker and continuous). To determine the agreement between channels, the respiratory phase pattern detected from the nasal channel was compared with that from the oronasal channel on the same swallowing trial. Within‐participant, within‐session variability (test–retest reliability) was calculated by comparing trial 1 and trial 2 of the same swallowing condition (e.g., spontaneous) from each channel (e.g., nasal or oronasal), from the same session (session 2).

The frequency of each respiratory phase pattern was descriptively analysed. The SNRF data were also descriptively analysed to determine the frequency of SNRF events and their position (onset‐pause and offset‐pause) and direction (inward or outward flow). Post hoc analyses were completed to assess the intra‐ and inter‐rater reliability of SNRF measurements. This was calculated using Cohen's unweighted κ (*P *= 0.05, 95% CI) and percentage of agreement. Due to the small sample of SNRF events, analyses were completed for all swallows combined (separated by nasal and oronasal channels) and were not separated by swallowing condition.

## 3 RESULTS

Twenty‐four participants (7 male) were recruited for the study. Four datasets were excluded from the analysis due to minor changes to the study protocol (3 datasets) and withdrawal from the study (1 dataset). A total of 280 swallowing trials were collected within the session 2 dataset, of which 41 were excluded due to problems with positioning of the facemask or sEMG electrodes. This left 239 swallows for statistical analysis.

### 3.1 Reliability

Intra‐ and inter‐rater reliability data (Cohen's κ and percentage of agreement) for the oronasal and nasal conditions are presented in Table 1. Overall, intra‐rater reliability was higher for the oronasal channel than for the nasal channel (across all swallowing conditions combined). Inter‐rater reliability was similar for both channels, although slightly higher for the nasal channel (κ = 0.73; *P* ≤ 0.001) than for the oronasal channel (κ = 0.68; *P* ≤ 0.001). When each swallowing condition was assessed separately, there was shown to be considerable variability across conditions. Intra‐rater reliability ranged from κ = 0.57–0.94 according to the the nasal channel and κ = 0.63–1 on the oronasal channel. Inter‐rater reliability ranged from κ = 0.44–0.94 for the nasal channel and κ = 0.43–0.84 for the oronasal channel. Reliability of the 10 mL swallows was consistently the lowest of all swallowing conditions.

**TABLE 1:** Intra‐ and inter‐rater reliability.

	Intra‐rater						Inter‐rater					
	Nasal			Oronasal			Nasal			Oronasal		
Swallowing conditions	Exact agreement (%)	Cohen's κ (95% CI)	*P*‐value	Exact agreement (%)	Cohen's κ (95% CI)	*P‐*value	Exact agreement (%)	Cohen's κ (95% CI)	*P‐*value	Exact agreement (%)	Cohen's κ (95% CI)	*P*‐value
All	84	0.78 (0.69, 0.86)	<0.001	87	0.81 (0.73, 0.89)	<0.001	81	0.73 (0.64, 0.82)	<0.001	79	0.68 (0.58, 0.78)	<0.001
Spontaneous	86	0.8 (0.62, 0.98)	<0.001	86	0.8 (0.63, 0.98)	<0.001	82	0.76 (0.58, 0.94)	<0.001	86	0.8 (0.63, 0.97)	<0.001
Dry	82	0.71 (0.49, 0.93)	<0.001	86	0.72 (0.47, 0.97)	<0.001	82	0.72 (0.52, 0.93)	<0.001	82	0.67 (0.4, 0.93)	<0.001
10 mL	68	0.57 (0.34, 0.79)	<0.001	75	0.63 (0.41, 0.85)	<0.001	57	0.44 (0.2, 0.67)	<0.001	61	0.43 (0.19, 0.67)	0.001
Continuous	89	0.84 (0.67, 1)	<0.001	100	1 (1, 1)	<0.001	86	0.8 (0.61, 0.99)	<0.001	89	0.84 (0.67, 1)	<0.001
Cracker	96	0.94 (0.83, 1)	<0.001	89	0.81 (0.63, 1)	<0.001	96	0.94 (0.84, 1)	<0.001	75	0.57 (0.33, 0.81)	<0.001

*Note*: Intra‐ and inter‐rater reliability is shown for the nasal and oronasal channels across all swallowing conditions combined, and separately, according to swallowing conditions. Cohen's κ (95% CI) = estimated Cohen's κ and 95% confidence interval (CI). Significance was taken as *P *= 0.05. Exact agreement (%) = percentage of exact agreement between ratings for each individual trial.

### 3.2 Comparison between oronasal and nasal data

Full Cohen's κ and percentage of agreement results are displayed in Table 2. Overall, agreement of respiratory phase patterns across the oronasal and nasal channels was estimated as κ = 0.75 (*P* ≤ 0.001), ranging from κ = 0.5 (10 mL; *P* ≤ 0.001) to κ = 0.91 (continuous; *P* ≤ 0.001).

**TABLE 2:** Agreement between nasal and oronasal channels.

Swallowing conditions (*n*)	Exact agreement (%)	Cohen's κ (95% CI)	*P*‐value
All (239)	81	0.75 (0.69, 0.81)	<0.001
Spontaneous (40)	90	0.87 (0.75, 0.99)	<0.001
Dry (100)	76	0.66 (0.55, 0.78)	<0.001
10 mL (40)	63	0.5 (0.32, 0.67)	<0.001
Continuous (60)	93	0.91 (0.83, 0.99)	<0.001
Cracker (40)	85	0.78 (0.63, 0.94)	<0.001

*Note*: Agreement between nasal and oronasal channels for all swallows combined and separated swallowing conditions. Cohen's κ (95% CI) = estimated Cohen's κ and 95% confidence interval. Significance was taken as *P *= 0.05. Exact agreement (%) = percentage of exact agreement between nasal and oronasal data.

### 3.3 Frequency of respiratory phase patterns across swallowing conditions and respiratory flow channels

The EX‐EX and IN‐EX patterns were the first and second most commonly occurring patterns, respectively, across both nasal and oronasal channels for all swallows combined. The EX‐EX pattern occurred in 42% (100) of swallows according to the nasal channel, increasing to 45% (107) from the oronasal channel. The IN‐EX pattern accounted for 27% (65) of swallows from the nasal channel, increasing to 30% (71) from the oronasal channel. The EX‐IN pattern also increased in frequency with the addition of oral flow, increasing from 11% (27) of swallows on the nasal channel to 13% (31) on the oronasal channel. In contrast, IN‐IN and CNJ trials both decreased in frequency with the addition of oral flow. The IN‐IN pattern accounted for 7% (16) of swallows (nasal), which decreased to 5% (13) of swallows from the oronasal channel. CNJ ratings decreased from 13% (31) of swallows (nasal) to 7% (17) of swallows (oronasal).

The frequencies of respiratory phase patterns according to the nasal and oronasal channels and across swallowing conditions are presented in Table 3 (see also Supplementary data). For spontaneous swallows, the two most common patterns were EX‐EX (36% nasal and 32% oronasal) and EX‐IN (32% nasal and oronasal), followed by IN‐EX (18% nasal and 25% oronasal). For cued dry swallows, the first and second most common patterns were EX‐EX (47% nasal and 49% oronasal) and IN‐EX (27% nasal and 29% oronasal), followed by EX‐IN (9% nasal and 11% oronasal). This was similar to continuous swallows, for which EX‐EX was most common (45% nasal and oronasal), IN‐EX was second most common (22% nasal and 24% oronasal), and EX‐IN was third most common (16% nasal and 18% oronasal). More than half of cracker swallows were EX‐EX (53% nasal and 63% oronasal), with most remaining swallows being IN‐EX (30% nasal and 23% oronasal). No cracker swallows were categorized as EX‐IN or IN‐IN on either the nasal or oronasal channels. Excluding CNJ, IN‐IN was the least common pattern for most respiratory phase categories, accounting for only 7% (nasal and oronasal) of spontaneous swallows, 4% (nasal) and 3% (oronasal) of cued dry swallows, and 8% (nasal) and 10% (oronasal) of continuous swallows. The exception to this was 10 mL swallows, for which IN‐IN (16% nasal and 8% oronasal) occurred more often than EX‐IN (5% nasal and 8% oronasal) swallows. In the 10 mL condition, IN‐EX was the most common pattern (39% nasal and 47% oronasal), followed by EX‐EX (21% nasal and 29% oronasal).

**TABLE 3:** Frequency of respiratory phase patterns.

Respiratory phase pattern	Conditions	Spontaneous	Cued dry	10 mL	Continuous	Cracker
		*n* (%)	*n* (%)	*n* (%)	*n* (%)	*n* (%)
EX‐EX	Nasal	10 (36)	43 (47)	8 (21)	23 (45)	16 (53)
	Oronasal	9 (32)	45 (49)	11 (29)	23 (45)	19 (63)
IN‐EX	Nasal	5 (18)	25 (27)	15 (39)	11 (22)	9 (30)
	Oronasal	7 (25)	27 (29)	18 (47)	12 (24)	7 (23)
EX‐IN	Nasal	9 (32)	8 (9)	2 (5)	8 (16)	0 (0)
	Oronasal	9 (32)	10 (11)	3 (8)	9 (18)	0 (0)
IN‐IN	Nasal	2 (7)	4 (4)	6 (16)	4 (8)	0 (0)
	Oronasal	2 (7)	3 (3)	3 (8)	5 (10)	0 (0)
CNJ	Nasal	2 (7)	12 (13)	7 (18)	5 (10)	5 (17)
	Oronasal	1 (4)	7 (8)	3 (8)	2 (4)	4 (13)

*Note*: Frequency (*n*) and percentage (%) of respiratory phase patterns across individual swallowing conditions according to nasal and oronasal conditions. Abbreviations: CNJ, cannot judge; EX‐EX, exhale–swallow–exhale; EX‐IN, exhale–swallow–inhale; IN‐EX, inhale–swallow–exhale; IN‐IN, inhale–swallow–inhale.

### 3.4 Within‐participant consistency of respiratory phase patterns

Across two trials of the same swallowing condition (completed in the same session), test–retest reliability of respiratory phase patterns ranged from 0.09 to 0.44 (nasal) and from 0.14 to 0.41 (oronasal). Across all conditions combined, within‐participant reliability of respiratory phase patterns was 0.28 (*P* ≤ 0.001) according to both the nasal and oronasal measures (Table 4).

**TABLE 4:** Within‐participant consistency across trials.

Swallowing conditions (*n*)	Nasal			Oronasal		
	Exact agreement (%)	Cohen's κ (95% CI)	*P*‐value	Exact agreement (%)	Cohen's κ (95% CI)	*P*‐value
All (84)	48	0.28 (0.12, 0.43)	<0.001	51	0.28 (0.13, 0.44)	<0.001
Spontaneous (28)	50	0.32 (−0.04, 0.67)	0.078	40	0.19 (−0.14, 0.52)	0.261
Dry (28)	47	0.21 (−0.07, 0.5)	0.199	58	0.41 (0.17, 0.66)	0.001
10 mL (28)	33	0.09 (−0.2, 0.38)	0.550	50	0.25 (−0.03, 0.53)	0.079
Continuous (28)	47	0.25 (−0.1, 0.6)	0.164	41	0.14 (−0.2, 0.49)	0.422
Cracker (28)	67	0.44 (0.04, 0.84)	0.030	60	0.25 (−0.17, 0.67)	0.241

Cohen's κ estimates and 95% confidence interval (95% CI) indicating consistency across two trials of the same consistency. Cohen's κ (95% CI)= estimated Cohen's κ and 95% confidence interval. Significance was taken as *P *= 0.05. Exact agreement = percentage of exact agreement between first and second trials of the same condition.

### 3.5 Swallowing non‐respiratory flow

SNRF was present during 49% (118) and 60% (144) of swallows across the nasal and oronasal channels, respectively. These occurred more commonly at the end of the respiratory‐swallowing pause (66% nasal and 62% oronasal) than at the beginning (34% nasal and 38% oronasal). SNRF appeared to occur at both onset‐pause and offset‐pause in 30% and 29% (nasal and oronasal) of the swallows where SNRF was present. Out of all SNRF events detected (337), almost all (99%) consisted of airflow moving in the opposite direction to the adjacent respiratory airflow, e.g., inward SNRF preceding exhalation. SNRF events stratified by respiratory phase pattern are presented in Table 5 (see also Supplementary data). Note that where a single swallow contained SNRF at both onset‐ and offset‐pause, these have been treated as two distinct events. Information regarding the reliability of SNRF data is available in Table 6.

**TABLE 5:** Swallowing non‐respiratory flow events stratified by respiratory phase pattern.

	EX‐EX	IN‐EX	EX‐IN	IN‐IN	Total
Position	*n* (%)	*n* (%)	*n* (%)	*n* (%)	
Onset pause (nasal)	10 (19)	32 (62)	7 (13)	3 (6)	52
Offset pause (nasal)	47 (47)	26 (26)	17 (17)	10 (10)	100
Onset pause (oronasal)	21 (30)	40 (57)	4 (6)	5 (7)	70
Offset pause (oronasal)	60 (52)	34 (30)	12 (10)	9 (8)	115

*Note*: Occurrences of SNRF events were stratified by respiratory phase pattern and position within the swallowing pause, for the nasal and oronasal channels. *n* = raw number of events; % = percentage out of all events per mask and condition. Total is the total number of SNRF events. Abbreviations: EX‐EX, exhale–swallow–exhale; EX‐IN, exhale–swallow–inhale; IN‐EX, inhale–swallow–exhale; IN‐IN, inhale–swallow–inhale; SNRF, swallowing non‐respiratory flow.

**TABLE 6:** Reliability of swallowing non‐respiratory flow data.

	Intra‐rater			Inter‐rater		
	Exact agreement (%)	Cohen's *k* (95% CI)	*P*‐value	Exact agreement (%)	Cohen's *k* (95% CI)	*P*‐value
Presence/absence	78.6	0.635 (0.518, 0.752)	<0.001	75	0.576 (0.455, 0.696)	<0.001
Position only	77.6	0.632 (0.480, 0.784)	<0.001	73.6	0.560 (0.398, 0.722)	<0.001
Direction only	75	0.509 (0.360, 0.658)	<0.001	78.8	0.562 (0.428, 0.696)	<0.001
Position and direction	73.7	0.653 (0.526, 0.781)	<0.001	65.3	0.544 (0.407, 0.681)	<0.001

*Note*: Cohen's κ estimates and 95% confidence interval (95% CI) indicating consistency across two trials of the same consistency. Cohen's κ (95% CI)= estimated Cohen's κ and 95% confidence interval. Significance was taken as *P *= 0.05. Exact agreement =  percentage of exact agreement within or between raters.

## 4 DISCUSSION

### 4.1 Intra‐ and inter‐rater reliability

Although sometimes high, agreement and reliability within‐ and between‐raters was variable according to both the nasal and oronasal channels; in particular, there was a lack of agreement and poor reliability on 10mL swallowing trials. This was most likely caused by an ambiguous respiratory waveform that was difficult to interpret. It is possible that the data were affected by between‐ or within‐participant behavioural inconsistencies. For example, it is possible that some of the instructions given to participants were unclear, leading to differences in ingestive behaviour. For the 10 mL water swallowing condition, the instruction, ‘swallow the water when you're readyʼ might have been interpreted differently by any given individual. For example, some participants might have swallowed the water as soon as it was presented, whereas others might have held the water in the mouth before swallowing. Such differences could have altered the recordings and subsequent respiratory or swallowing measures. Stricter instructions may be of use in future studies to encourage more consistent swallowing behaviour across participants. There also appeared to be disagreement between raters for the cracker trials according to the oronasal mask. The large difference in inter‐rater reliability of cracker swallows using the nasal and oronasal channels might have resulted from artefact in the respiratory signal caused by movement of the facemask during chewing. It is feasible that jaw movement would have impacted the oral compartment of the mask to a greater extent than the nasal compartment, resulting in more disruption to the respiratory waveform.

### 4.2 Frequency of respiratory phase patterns

When all swallowing conditions were combined, EX‐EX was the most common respiratory phase pattern according to both the nasal and oronasal channels. This is consistent with findings from a previous meta‐analysis indicating EX‐EX predominance (Hopkins‐Rossabi et al., 2019). However, the frequency of EX‐EX swallows in this study was considerably reduced compared with previous values (Hopkins‐Rossabi et al., 2019; Martin‐Harris et al., 2005; Wheeler Hegland et al., 2009), with higher occurrence of EX‐IN, IN‐EX and IN‐IN swallows than previously reported (Martin‐Harris et al., 2005; Wheeler Hegland et al., 2009). One possible explanation is that the present study included a larger range of swallowing conditions, across which the frequency of each respiratory phase pattern would be expected to vary due to differences such as bolus volume (Hopkins‐Rossabi et al., 2019; Lederle et al., 2012) or use of a straw (Wheeler Hegland et al., 2011). It is also possible that the sealed oronasal facemask detected respiratory parameters differently to nasal prongs and/or kinematic measures. Additionally, the more restrictive and less natural oronasal facemask method might have altered the breathing and swallowing behaviour of participants, causing differences in findings. A more in‐depth investigation of respiratory physiology across swallowing tasks is required to determine the validity of the oronasal facemask for examining respiratory phase patterns.

Findings from various ingestive (10 mL water, continuous water and cracker) and non‐ingestive (spontaneous and cued dry) swallowing conditions in the present study indicate variability in the frequency of respiratory phase patterns across tasks. These findings highlight the importance of comparing a range of swallowing conditions, including spontaneous and cued non‐ingestive swallows, as well as ingestive swallows from a range of bolus types, volumes and delivery methods. It is clear that 10 mL water swallows require further investigation due to the findings of low reliability and agreement in the present study. However, it is possible that these findings were influenced by the equipment that was used or by the instructions that were given for each condition; these factors require further investigation.

### 4.3 Influence of oral flow on respiratory phase patterns

Inclusion of oral respiratory data minimally altered respiratory phase pattern estimates in a healthy cohort, with EX‐EX, IN‐EX and EX‐IN patterns increasing by 2%–3% compared to isolated nasal flow. In contrast, the IN‐IN and CNJ patterns decreased in frequency with the addition of oral flow. It is likely that inclusion of oral flow increased the clarity of the respiratory waveform, therefore improving the raters' ability to determine the respiratory phase pattern. This is supported by the finding that the CNJ category had the largest reduction in frequency when oral flow was added. Previously, Hopkins‐Rossabi et al. (2020) reported that unambiguous nasal airflow (measured with a nasal cannula) occurs above ±0.028 cm H_2_O. In the present study, it is possible that nasal flow was below the threshold for swallows categorized as CNJ, and that the additional oral flow increased the combined pressure signal above the threshold for detection. It is likely that nasal‐only estimates of RSC in healthy populations are not vastly different than what would be seen with inclusion of oral flow. However, the difference between nasal and oronasal respiratory estimates may be larger in populations prone to mouth breathing, such as chronic cough (Vertigan et al., 2020) and sleep apnoea (Koutsourelakis et al., 2006). Assessment of these populations is required to confirm this hypothesis. Observations from our dataset suggest that individuals sometimes switched between oronasal and nasal‐only breathing, supporting previous reports of variable respiratory routing (Fujimoto et al., 2009). Whether switching between oronasal and nasal‐only respiration impacted RSC was not directly assessed, though this may be of interest in future investigations.

Several studies have indicated that respiration continues during oral bolus preparation (Charbonneau et al., 2005; Matsuo & Palmer, 2008). Other research has indicated that many individuals pause respiration (as measured with a nasal cannula) at the time of bolus transfer into the mouth (Martin‐Harris et al., 2005). Ingestive swallowing requires closure of the mouth for bolus containment, and for facilitating a closed system for pressure build up. A limitation of this study is that combining nasal and oral flow into one channel (oronasal) prohibited assessment of temporal differences in the cessation of flow between the nasal‐only and oral‐only channels. Depending on whether breathing continues through the nose during the oral preparatory phase, a mouth‐breathing individual may show different flow parameters (i.e., timing of onset and offset of flow) across the oral and nasal channels, with respiratory flow still detectable at the nose after closing the mouth. Directly comparing the nasal‐only and oral‐only channels may more clearly demonstrate respiratory routing and upper airway biomechanics in preparation for swallowing, particularly in mouth breathers. Assessing oral flow separately may also demonstrate longer pauses in airflow on the isolated oral flow channel compared to nasal or oronasal channels, supported by observations of this phenomenon in mouth breathers in the current dataset. However, this observation was limited because the timing of the respiratory‐swallowing pause was not objectively measured.

Agreement analyses suggest that the addition of oral airflow altered measurement of respiratory phase patterns to varying degrees across swallowing tasks, with some conditions being more likely to be altered by its inclusion than others. The higher agreement between nasal and oronasal channels for the spontaneous and continuous conditions suggests that adding oral flow was of little influence. A larger difference was seen between channels on cued dry, cracker and 10 mL trials, suggesting that inclusion of oral flow had a greater impact. This was particularly pertinent for 10 mL swallows; however, the 10 mL trial results should be interpreted with added caution due to the reduced intra‐ and inter‐rater reliability observed in this condition. More in‐depth physiological investigations are required to ascertain the reason for these differences and to determine the clinical importance of considering oral respiration in RSC investigations.

### 4.4 Within‐subject, within‐session reliability

The findings from this study suggest that individuals may behave inconsistently regarding respiratory phase patterning. This finding is supported by previous reports of participants using multiple respiratory phase patterns across trials (Hardemark Cedborg et al., 2009; Wheeler Hegland et al., 2011, 2009). For example, one study reported that 70% of participants used all four respiratory phase patterns, and the rest of the cohort used more than one pattern across continuous drinking trials (Wheeler Hegland et al., 2011). In a different study assessing discrete bolus trials (water and paste), all participants used the EX‐EX pattern on at least one occasion, while 55% demonstrated an IN‐EX pattern, 35% used an IN‐IN pattern, and 80% used the EX‐IN pattern (Wheeler Hegland et al., 2009). In another study, during spontaneous saliva swallows, one third of the sample used both EX‐EX and EX‐IN patterns across trials (Hardemark Cedborg et al., 2009). The results of the present study and several previous studies (Hardemark Cedborg et al., 2009; Wheeler Hegland et al., 2011, 2009) are contradicted by other findings of relative consistency across trials, with 71%–75% of swallows following EX‐EX patterning over two trials (Martin‐Harris et al., 2005). However, although the prevalence of EX‐EX swallows was relatively consistent across trials (Martin‐Harris et al., 2005), this was calculated for the entire group, and a within‐participant test‐ retest analysis was not reported. Therefore, the true variability of respiratory phase patterns across trials within the same participant remains unknown.

The low within‐subject reliability of respiratory phase patterns in the present study might have been influenced by the use of a fully enclosed oronasal facemask by decreasing the naturalness of breathing and swallowing tasks. Additionally, the lack of certainty regarding what constitutes a SNRF event might have affected respiratory phase pattern analyses, therefore altering estimation of respiratory phase patterns across trials. The way in which the oronasal facemask measured airflow is likely to have altered the presentation of SNRF, with its increased sensitivity to capture all flow, therefore affecting the frequency of each respiratory phase pattern, intra‐ and inter‐rater reliability, and subsequently, within‐subject variability. Further investigation is needed to determine the validity of the oronasal facemask for assessment of RSC before conclusions can be drawn regarding the findings of the present study. It is also possible that these findings were influenced by artefact in the spirometry signal, e.g., caused by chewing during the cracker tasks or by pressure changes created by the straw during drinking tasks. Although somewhat unavoidable when using an oronasal facemask to assess ingestive swallowing, the impact of these tasks on the respiratory signal should not be underestimated until they are better understood. Future studies should use a stopcock when presenting the bolus through a straw to reduce the effect of intra‐straw airflow or pressure.

It would be beneficial to assess the consistency of respiratory phase patterns within participants across an increased number of trials of each swallowing condition. In the present study, we assessed within‐subject reliability across two trials of each swallowing condition, suggesting a lack of consistency. Increasing the number of trials might provide insight into whether this variability follows a pattern. For example, it might be normal and safe for an individual to switch consistently between two patterns, such as EX‐EX and IN‐EX. Such a pattern could be elucidated more clearly across an increased number of trials. Improved understanding of respiratory phase pattern variability in healthy subjects is of great importance for establishing the clinical significance of respiratory phase patterns and for determining the clinical utility of targeted RSC treatments in dysphagic individuals.

### 4.5 Swallowing non‐respiratory flow

Findings from the present study suggest that SNRF might present more variably than previously reported (Brodsky et al., 2012). In addition to previous research, which has detected non‐respiratory (non‐inspiratory) flow events at the end of the respiratory‐swallowing pause (Brodsky et al., 2012; Martin‐Harris et al., 2003; Paydarfar et al., 1995), this study has identified extraneous flow at the onset of the pause, in addition to apparent non‐respiratory outward (non‐expiratory) airflow. It is likely that the instrumentation used for respiratory measurement has contributed to this finding. It is feasible that the oronasal facemask provides more sensitive airflow measures than the nasal cannula method on account of it being fully sealed. It is unlikely that the change in SNRF presentation was attributable to the addition of oral flow, because we would expect the mouth to be closed immediately before, during, and after each swallow. However, findings demonstrate that SNRF occurred more frequently on the oronasal channel, with an increase of 11%. It is also possible that these findings of more variable SNRF events might also be the product of artefact caused by the oronasal facemask, rather than a true reflection of non‐respiratory pressure changes. Further investigations are required before definitive conclusions can be drawn about these findings. Respiratory measurements taken from abdominal and rib cage movement simultaneously with oronasal flow would likely assist with distinguishing between true respiratory flow and non‐respiratory flow to confirm whether these oronasal measures reflect distinct SNRF events.

Given that judging the respiratory phase surrounding swallowing is theoretically relatively objective, it is likely that the poor intra‐ and inter‐rater reliability seen in some conditions was caused by disagreement regarding what does or does not constitute SNRF, therefore changing interpretation of the respiratory phase pattern. This theory is supported by post hoc findings of within‐ and between‐rater disagreement regarding what constitutes an SNRF event. These findings indicate that the raters were unable to distinguish consistently between SNRF and respiratory flow. It is likely that signal artefact also contributed by creating further ambiguity in the waveform. The difficulties encountered during data analysis were impacted, in part, by the differences in SNRF in this study in comparison to others. For example, SNRF observed using spirometry appears to present in a slightly different manner visually compared with the characteristic swallowing non‐inspiratory flow (SNIF) reported previously (Brodsky et al., 2012; Martin‐Harris et al., 2003). Examples of SNRF events from our dataset can be seen in Figures 1 and 2. Some variation existed in the shape of the SNRF waveform across trials, sometimes making it difficult to determine whether the event was respiratory, non‐respiratory or an artefact. Additionally, the lack of data regarding amplitude and duration of SNRF results in a lack of guidance for raters. Although temporal data have been published previously (Brodsky et al., 2012), these were related to the timing of SNRF in relationship to other physiological swallowing events, rather than describing the SNRF itself. Standard operating procedures published since completion of this study suggest that a duration of >0.246 s is an appropriate threshold (Curtis & Borders, 2024), based on previous data from the study by Paydarfar et al. (1995). These new guidelines are an important step in standardizing analysis of SNRF. However, there is still a need for targeted research to determine the expected amplitude and duration of a SNRF event. These values will potentially vary across measurement tools. An improved definition of the duration and amplitude of SNRF events would be of great assistance in standardizing RSC research by providing clarity regarding what constitutes SNRF.

## AUTHOR CONTRIBUTIONS

Elizabeth Cross and Phoebe Macrae conceptualized and designed the study, and Esther Guiu Hernandez designed the statistical approach. Data were collected and analysed by Elizabeth Cross and Phoebe Macrae, and statistically analysed by Esther Guiu Hernandez. Interpretation of data was conducted by Elizabeth Cross, Esther Guiu Hernandez and Phoebe Macrae. The manuscript was drafted by Elizabeth Cross, with revisions completed by Esther Guiu Hernandez and Phoebe Macrae. All authors approved the final version of the manuscript and agree to be accountable for all aspects of the work in ensuring that questions related to the accuracy or integrity of any part of the work are appropriately investigated and resolved. All persons designated as authors qualify for authorship, and all those who qualify for authorship are listed.

## CONFLICT OF INTEREST

None declared.

## FUNDING INFORMATION

None.

## Supporting information

Supporting data demonstrating respiratory phase patterns for each swallowing trial, according to nasal and oronasal channels; SNRF data for each swallowing trial, according to nasal and oronasal channels; and test–retest reliability data according to nasal and oronasal channels.

## Data Availability

Raw data have been made available in the supporting spreadsheet. These include the respiratory phase patterns for each trial according to the nasal and oronasal channel, presence/absence and direction of SNRF events for each trial according to nasal and oronasal data, and test–retest reliability of respiratory phase patterns.
